# Reciprocal co-regulation of nitrate and ammonium transporters is modulated by external pH in Arabidopsis

**DOI:** 10.1093/jxb/erag007

**Published:** 2026-01-13

**Authors:** Mikel Rivero-Marcos, Nicolaus von Wirén

**Affiliations:** Molecular Plant Nutrition, Leibniz Institute of Plant Genetics and Crop Plant Research (IPK), Corrensstrasse 3, D-06466 Gatersleben, Germany; Molecular Plant Nutrition, Leibniz Institute of Plant Genetics and Crop Plant Research (IPK), Corrensstrasse 3, D-06466 Gatersleben, Germany; Universität zu Köln, Germany

**Keywords:** Ammonium, ammonium uptake, AMTs, high-affinity nitrate uptake, nitrate, NRT1.1, NRT2.1, NSCC

## Abstract

Plants primarily acquire inorganic nitrogen (N) as nitrate (NO_3_^−^) and ammonium (NH_4_^+^). The uptake of these forms is strongly modulated by external pH, which influences both their availability and the activity of their specific root transporters (NRTs for NO_3_^−^ and AMTs for NH_4_^+^). Moreover, NO_3_^−^ and NH_4_^+^ uptake exerts opposite effects on net proton (H^+^) fluxes, raising the question of how external H^+^ availability shapes the balance between both N forms and their reciprocal regulation. Using Arabidopsis knockout mutants deficient in key NO_3_^−^ and NH_4_^+^ transporters, in combination with N and H^+^ flux assays and gene expression analyses, this study shows that low external pH strongly promotes NO_3_^−^ uptake but severely constrains plant growth under NH_4_^+^ nutrition. The stimulatory effect of external H^+^ over-rides the H^+^ efflux typically induced by NH_4_^+^. Conversely, at higher external pH, an alternative, AMT-independent transport mechanism probably related to K^+^ transport appears to facilitate NH_4_^+^ uptake and mitigate its toxicity. Furthermore, mutants lacking AMTs exhibited enhanced high-affinity NO_3_^−^ uptake at low pH, while the NRT1.1 mutant (*chl1-5*) showed increased high-affinity NH_4_^+^ acquisition at higher pH. These findings highlight a new and complex interplay between pH and reciprocal N uptake dynamics and point to AMT- and NRT1.1-independent pathways contributing to the acquisition of alternative N forms under contrasting pH conditions.

## Teaser

External pH governs nitrate and ammonium uptake balance in Arabidopsis, revealing novel reciprocal regulation of their transporters that shape nitrogen acquisition beyond classical transport functions.

## Introduction

In quantitative terms, nitrogen (N) is the most important mineral element for crops, acquired primarily in the form of ammonium (NH_4_^+^) and nitrate (NO_3_^−^). It has long been recognized that low external pH promotes NO_3_^−^ uptake, because secondary active transport of NO_3_^−^ across the plasma membrane (PM) of roots is coupled to the co-transport of two H⁺ and leads to alkalization of the apoplast and rhizosphere (McClure *et al*., 1990; Tsay *et al*., 1993; Kosegarten *et al*., 1999). In the presence of NH_4_^+^, however, NH_4_^+^ uptake-dependent H^+^ secretion compensates for H^+^ consumption by NO_3_^−^ uptake, allowing roots to balance their cation–anion uptake ratio and decrease the energy investment for compensatory H⁺ fluxes. The uptake of NH_4_^+^ itself is also affected by external pH, with increasing influx at near-neutral to alkaline pH, where the increasing proportion of free ammonia (NH_3_) can diffuse across membranes more easily (Downing and Merkens, 1955; Kleiner, 1985).

In well-aerated agricultural soils, NO_3_^−^ is the predominant N form, with concentrations in the soil solution typically ranging from 1 mM to 5 mM, while NH_4_^+^ concentrations may range from <20 μM to 200 μM (Lark *et al*., 2004). In contrast to NO_3_^−^ that is susceptible to leaching, NH_4_^+^ can be adsorbed by the soil matrix and remain plant available through subsequent desorption, acting as a slow-release process (Muratore *et al*., 2021). In acidic or paddy soils, NH_4_^+^ can accumulate up to millimolar concentrations, especially after NH_4_^+^ fertilization, which can even hinder plant growth (Xu *et al*., 2012). With regard to the strongly changing availabilities of NH_4_^+^ and NO_3_^−^ in soils with different pH, the question arises of how plants maintain their overall cation–anion uptake balance, which is dominated >70% by NH_4_^+^ and NO_3_^−^ uptake (Kirkby and Mengel, 1967), and whether compensatory H^+^ fluxes are sufficient.

In addition to steering the availability of NH_4_^+^ and NO_3_^−^, soil pH interferes with the regulation of the PM-localized dual-affinity NRT1.1 and high-affinity NRT2-type NO_3_^−^ transporters. Irrespective of the presence of NO_3_^−^, lowering the external pH from 6.5 to 5.5 led to increased *NRT1.1* gene expression (Tsay *et al*., 1993; Fang *et al*., 2016), suggesting that pH alone can trigger *NRT1.1* induction and thereby increase NRT1.1-mediated repression of the high-affinity transporter gene *NRT2.1*. However, it remains unclear whether binding of H^+^ to NRT1.1 is also required for its sensing function and its repressive effect on *NRT2.1* transcript levels (Ho *et al*., 2009; Parker and Newstead, 2014; Sun *et al*., 2014).

With respect to NRT2.1-mediated NO_3_^−^ transport activity, low pH significantly decreased C-terminal phosphorylation of the NO_3_^−^ transporter NRT2.1, particularly at T521, a regulatory mechanism increasing its transport activity (Jacquot *et al*., 2020; Jain and Schmidt, 2024). This apparently contradicts the observation that *NRT2.1* expression is repressed in response to acidic pH (Bailey *et al*., 2023) and suggests an opposite pH-dependent transcriptional regulation of *NRT1.1* and *NRT2.1*, although both high- and low-affinity NO_3_^−^ transporters depend on H^+^ co-transport.

On the contrary, among NH_4_^+^ transporters, only the transport activities of AMT1.1 in wheat and common bean have been shown to be pH dependent, exhibiting an acid-stimulated regulatory mode (Søegaard, 2009; Ortiz-Ramirez *et al*., 2011), whereas the transport activities in Arabidopsis, tomato, and rice are pH independent when expressed in oocytes (Ludewig *et al*., 2002, 2003; Loqué *et al*., 2006, 2007; Wood *et al*., 2006; Yang *et al*., 2015). However, to date, there are no clear studies on how the AMTs may contribute to NH_4_^+^ uptake in response to external pH variations, particularly considering their physiological relevance to plants.

Given this scenario, in which low or high pH conditions appear to preferentially recruit NRT1.1 or NRT2.1, respectively, for high-affinity NO_3_^−^ uptake, the following is hypothesized: (i) external pH will be the key determinant of NO_3_^−^ uptake capacity; (ii) consequently, the enhanced H^+^ secretion in response to AMT-dependent NH_4_^+^ uptake will further stimulate NO_3_^−^ uptake; and (iii) this low pH-promoted NO_3_^−^ uptake may, in turn, favor NH_4_^+^ uptake capacity, thereby contributing to the balance of anion and cation uptake.

Since AHA2 has been described as a major PM H^+^-ATPase in roots responsible for H^+^ secretion, especially in response to NH_4_^+^ uptake (Meier *et al*., 2020), we examined here pH-dependent high-affinity NO_3_^−^ and NH_4_^+^ influx in plants defective in AHA2 (*aha2*), NRT1.1 (*chl1-5*), and AMT transporters (*qko*). To diff4erentiate the impact of the NH_4_^+^-to-NO_3_^−^ uptake ratio on external H^+^ balance from the impact of external pH, the nutrient solution was buffered to different pH levels to investigate the uptake of each N form in the absence or presence of the other. Since K^+^ is known to stabilize the electrochemical gradient across membranes and counteract imbalances in the cation–anion uptake ratio, we also used Rb^+^ as a tracer for K^+^ and measured its influx depending on the N form and pH under the same experimental conditions. Collectively, these findings challenge the assumption of a balanced cation–anion uptake over total N acquisition, particularly when either NO_3_^−^ or NH_4_^+^ transport is disturbed.

It should be noted that while the use of a buffered hydroponic system is an artificial condition, this reductionist approach was a prerequisite to experimentally decouple the effects of N nutrition from the confounding influence of external pH, an objective that would be unattainable in a complex soil matrix.

## Materials and methods

### Plant material and growth conditions

The *Arabidopsis thaliana* L. wild-type ecotype used in this study was Col-0. The following mutants and transgenic lines were used: quadruple knockout line *qko* that is defective in four root-expressed NH_4_^+^ transporter genes (*amt1.1*, *amt1.2*, *amt.1.3*, and *amt2.1*; Yuan *et al*., 2007); *nrt1.1* (*chl1-5*; Tsay *et al*., 1993), and *aha2-4* (Haruta *et al*., 2010).

Arabidopsis seeds were first germinated on rock wool moistened with tap water for 1 week. During the second and third weeks, seedlings were transferred to 5 liter hydroponic pots, containing half-strength, and subsequently full-strength, nutrient solution, of the following composition: 1 mM KH_2_PO_4_, 1 mM MgSO_4_, 250 μM K_2_SO_4_, 250 μM CaCl_2_, 100 μM Na-Fe-EDTA, 50 μM KCl, 50 μM H_3_BO_3_, 5 μM MnSO_4_, 1 μM ZnSO_4_, 1 μM CuSO_4_, and 1 μM NaMoO_4_. Ca(NO_3_)_2_ at 0.5 mM was supplied to provide N-sufficient conditions. In the fourth week, the treatments were differentiated and the seedlings received one of three N treatments: 2.5 mM Ca(NO_3_)_2_, 2.5 mM (NH_4_)_2_SO_4_, or 1.25 mM of both salts. The (NH_4_)_2_SO_4_ treatment was supplemented with 2.5 mM CaSO_4_. Each N treatment was buffered with 5 mM MES/Tris at pH 4.5, 5.5, or 6.5. For each treatment, six plants per line were grown in a hydroponic pot, with three independent pots per combination of external pH and N regime. Each individual plant was considered a biological replicate. The nutrient solution was replaced every 3 d. Plants were grown for 6 weeks until they reached growth stage 5.10 according to Feller *et al*. (1995). The plants were cultivated in a climate-controlled growth chamber under a 10 h/14 h and 22 °C/18 °C light/dark cycle, a light intensity of 240 μmol m^−2^ s^−1^, and 70% relative humidity. To account for diurnal variations in gene expression and N uptake, all samples were harvested at a fixed time point, 2–3 h after onset of the light period.

For the analysis of cytosolic acidification, we used the transgenic line Pt-GFP in the Col-0 background (Nottingham Arabidopsis Stock Centre, N9561), expressing the pH reporter protein Pt–green fluorescent protein (GFP) in the cytosol. Seeds were surface-sterilized with 70% ethanol containing 0.05% Triton X-100, followed by a 10% bleach treatment and several rinses with sterile water, and then stratified in the dark at 4 °C for 2 d to synchronize germination. For pre-culture, seeds were sown on Petri dishes containing half-strength Murashige and Skoog (1/2 MS) medium supplemented with 1% (w/v) sucrose and solidified with 1% (w/v) agar, with pH adjusted to 5.7 using MES buffer. Seedlings were grown vertically for 5 d in a controlled-environment chamber under a 16 h/8 h light/dark period at 22 °C. After pre-culture, seedlings of uniform size were transferred to square plates containing 1/2 MS medium without N, buffered with MES to pH 4.5, 5.5, or 6.5, and supplied with different N sources: 5 mM Ca(NO_3_)_2_, 5 mM (NH_4_)_2_SO_4_, or 1.25 mM of each salt for the mixed-N treatment.

### Cytosolic pH imaging and analysis

Whole seedlings were carefully transferred from the agar plates to a 35 mm glass-bottom imaging dish. Confocal microscopy was performed using a CLSM-Zeiss 780 system with a ×20 water-immersion objective. To visualize cytosolic pH changes, Pt–GFP was excited at 476 nm, and fluorescence emission was recorded at 500–540 nm (green channel) (Schulte *et al*., 2006). Pt–GFP fluorescence intensities were quantified in the root apical meristem and differentiation zone, and the shoot apical meristem after background subtraction. To provide a structural reference and clearly define the cell boundaries of the root and shoot zones, seedlings were counterstained with 10 µg ml^−1^ propidium iodide (PI) for 5 min. The resulting 12-bit images were analyzed using ImageJ. Because Pt–GFP responds ratiometrically, higher ratios correspond to more acidic cytosolic pH.

### ^15^N uptake

Influx of ^15^N-labeled NH_4_^+^ or NO_3_^−^ into plant roots was measured in 6-week-old hydroponically grown plants. Roots were rinsed with CaSO_4_ for 1 min in order to saturate the cation exchange capacity in the apoplast and stabilize the PM. Plants were then transferred to a buffered nutrient solution containing 0.2 mM ^15^N-labeled NH_4_^+^ or NO_3_^−^ [≥98 atom % ^15^N as (NH_4_)_2_SO_4_ or Ca(NO_3_)_2_, respectively] at pH 4.5, 5.5 or 6.5, for a period of 10 min. To assess the effect of a greater dose of NO_3_^−^ on high-affinity NH_4_^+^ uptake, 0.2 mM ^15^N-labeled NH_4_^+^ was supplied in combination with non-labeled 5 mM NO_3_^−^. Conversely, to evaluate the effect of a greater dose of NH_4_^+^ on high-affinity NO_3_^−^ uptake, 0.2 mM ^15^N-labeled NO_3_^−^ was provided together with non-labeled 5 mM NH_4_^+^. Subsequently, the roots were rinsed with 1 mM CaSO_4_ for 1 min to remove the tracer from the apoplast. Roots were sampled and stored at −80 °C before freeze-drying. Ground samples were used for [^15^N]isotope and total N analysis by isotope ratio mass spectrometry (IRMS; Horizon, NU Instruments).

### Determination of H^+^ efflux from roots

To measure H^+^ flux, roots of 6-week-old plants were immersed for 5 h under light in a MES-free nutrient solution. The solution was maintained at a stable volume-to-FW ratio of 100:1 (100 ml of solution per g of root FW; modified from Hachiya *et al*., 2021). Subsequently, changes in pH were converted to changes in H^+^ concentrations by [H^+^]=10^−pH^, and expressed as the flux rate of H^+^ in µmol h^−1^ g^−1^ FW. The pH of the hydroponic culture was monitored daily from the fourth week on, when seedlings had developed sufficient root biomass to reliably record pH changes. The pH in the hydroponic solution was measured using a pH meter (B212, Horiba, Ltd), and each of the three pots per treatment and line was considered a replicate.

### Real-time quantitative PCR

Root material consisted of 6-week-old plants that were pre-cultured under the above-mentioned N and pH regimes to assess steady-state gene expression levels. Total RNA was extracted using phenol:chloroform:isoamyl alcohol (25:24:1 v/v/v). The resulting RNA-containing aqueous phase underwent precipitation and purification. To ensure the complete removal of genomic DNA, an on-column DNase I digestion was performed using a commercial kit (Sigma-Aldrich, Cat. No. DNAse10). RNA quality was determined using NanoDrop™ 2000c spectrophotometry (Thermo Scientific™). A 2 μg aliquot of total RNA was used for cDNA synthesis using the RevertAid First Strand cDNA Synthesis Kit (Thermo Scientific) and oligo(dT)_12–18_ primers. Real-time PCR was conducted on a Mastercycler ep realplex (Eppendorf) using QuantiTect SYBR Green qPCR mix (Bio-Rad Laboratories, Hercules, CA, USA). Primer specificity was validated through melting curve analysis. Relative expression levels were calculated following the method of Pfaffl (2001). For the selection of a reliable internal control, we compared the expression stability of three candidate reference genes: ubiquitin 10 (*UBQ10*, At4g05320), actin 2 (*ACT2*, At3g18780), and glyceraldehyde-3-phosphate dehydrogenase (*GAPDH*, At1g13440). Analysis of the raw cycle threshold (Ct) values across our complete sample set revealed that *UBQ10* displayed significantly lower variability (SD=0.06) compared with both *ACT2* (SD=2.82) and *GAPDH* (SD=1.76). Due to its superior expression stability, *UBQ10* was chosen as the reference gene for normalization of all real-time quantitative PCR data (Supplementary Table S1). The gene-specific primer pairs used in this study are detailed in Supplementary Table S2.

### Rb^+^ uptake

Similar to as described for ^15^N uptake studies, six plants of each treatment were transferred to 1 mM CaSO_4_ for 1 min, followed by incubation in 1 mM RbCl for 10 min, before transfer for 1 min to 1 mM CaSO_4_ to wash out Rb from the root apoplast. Rb influx was calculated by referring the Rb concentration to root DW.

### Statistical analysis

All experiments were repeated independently with at least three biological replicates. Statistical analyses were conducted using GraphPad Prism version 10.4. For comparisons between wild-type and individual mutants with normally distributed data, Student’s *t*-test was applied. For comparisons among three or more groups, one-way ANOVA followed by Tukey’s post-hoc test was used. Statistical significance was defined at *P*<0.05. Detailed statistical methods for each experiment are provided in the respective figure legends.

## Results

### Impact of nitrogen forms and external pH on root and shoot development

Since nutrient uptake capacities strongly depend on the physiological state of the plant, an initial growth experiment examined the influence of external pH and supplied N forms on biomass formation and root development. As expected, shoot biomass formation was highest under sole NO_3_^−^ supply or mixed N nutrition, irrespective of the pH in the growth medium (Supplementary Fig. S1A). In all lines, sole NH_4_^+^ nutrition caused a decrease in shoot biomass, which is typical for NH_4_^+^ toxicity and a consequence of excess acidity accumulating in the shoot and being relieved by increasing external pH (Hachiya *et al*., 2021). In comparison, depression of root biomass and root elongation by sole NH_4_^+^ nutrition was relatively weaker, except at pH 4.5 when root biomass and length were lowest across all treatments (Supplementary Fig. S1B, C). Such impaired root development was associated with cytosolic acidification (Supplementary Fig. S2) and particularly pronounced in the *aha2* mutant, probably due to weaker H^+^ secretion. The strongest suppression of shoot and root biomass under exclusive NH_4_^+^ nutrition occurred in *qko*, where increasing the medium pH failed to alleviate NH_4_^+^-dependent growth inhibition as effectively as in the other lines.

In the presence of NO_3_^−^, shoot biomass was lowest in the *chl1-5* mutant, underscoring the importance of NRT1.1 for bulk NO_3_^−^ uptake and N provision of the plant. Hence, N concentrations in the root tissue were slightly lower but still considered adequate (Supplementary Fig. S3). By contrast, root N levels of the other lines were even higher at low pH, probably also reflecting NO_3_^−^ storage in the root. Nonetheless, all lines had adequate root N concentrations, confirming that N deficiency played no role in growth and N uptake behavior in any of the treatments. Notably, under NO_3_^−^ supply, root biomass of the *aha2* mutant appeared to be less compromised by pH 4.5, indicating that a lack of H^+^ secretion by AHA2 alleviates H^+^ stress, even under NO_3_^−^ nutrition. Altogether, this growth experiment showed that (i) root development in particular is impaired at pH 4.5, irrespective of N form; (ii) in the presence of NH_4_^+^, lack of AHA2-dependent H^+^ secretion further impairs root development; and (iii) co-supply of NO_3_^−^ alleviates the growth-suppressive effects of NH_4_^+^, rendering root and shoot development highly similar to that of plants supplied only with NO_3_^−^.

### Role of external H^+^ availability on gene expression of NO_3_^−^ transporters and NO_3_^−^ influx

**Fig. 1.**
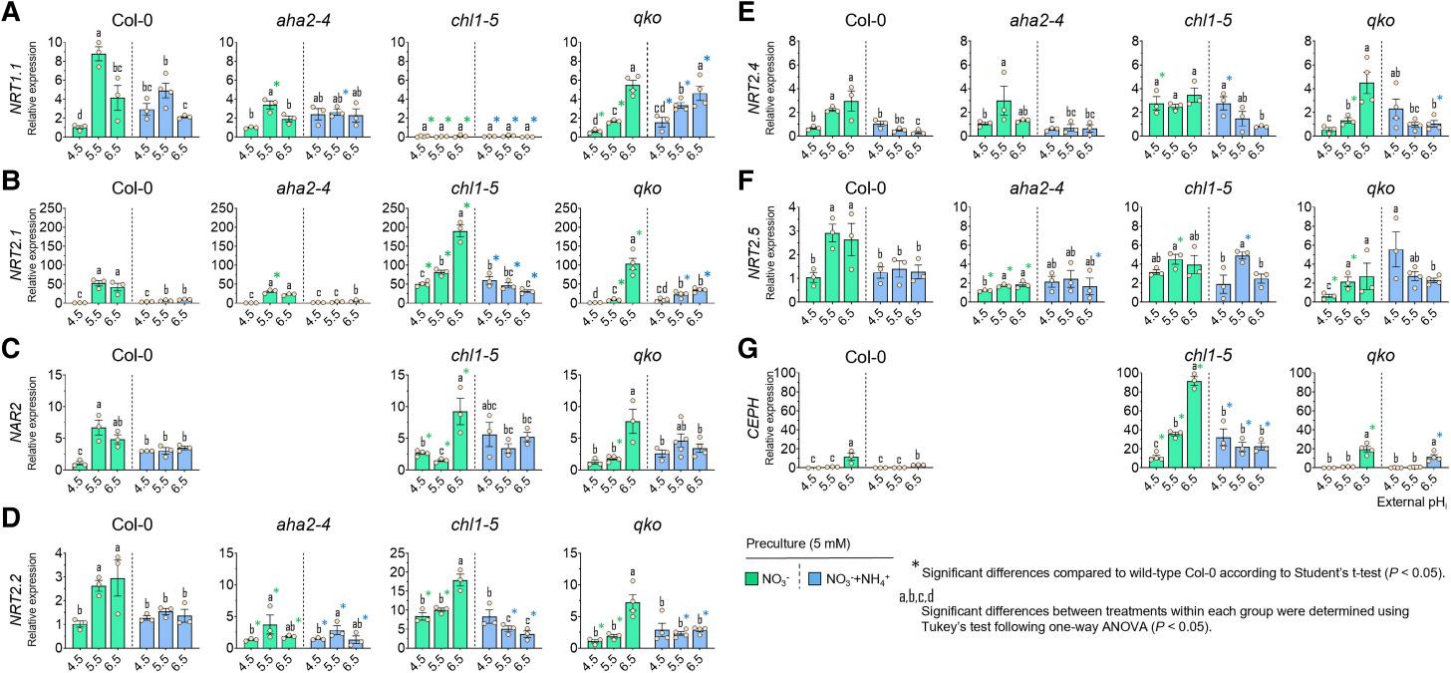
NH_4_^+^ uptake moderates the transcriptional induction by higher pH of the major high-affinity NO_3_^−^ transporters, although it does not explain the pH-dependent pattern of NO_3_^−^ uptake. Relative transcript levels of *NRT1.1* (A), *NRT2.1* (B), *NAR2* (C), *NRT2.2* (D), *NRT2.4* (E), *NRT2.5* (F), and *CEPH* (G) were quantified by RT–qPCR in roots of 6-week-old Arabidopsis wild-type (Col-0) plants and the mutant lines *aha2-4*, *chl1-5*, and *qko*. Plants were precultured with 5 mM NO_3_^-^ and subsequently treated with NO_3_^-^ alone or NO_3_^-^ plus NH_4_^+^ at external pH 4.5, 5.5, or 6.5. Transcript abundance was normalized to *UBQ10*. Data represent means ± SE (*n* = 3–4). Different letters indicate significant differences among treatments within each genotype (one-way ANOVA followed by Tukey’s test, *P* < 0.05). Asterisks denote significant differences compared with Col-0 within the same pH treatment (Student’s *t*-test, *P* < 0.05).

Before we examined N uptake, we recorded to what extent the two different pre-cultures affected transcript levels of the most relevant NO_3_^−^ transporters. In Col-0, transcript levels of *NRT1.1* were highest at pH 5.5 and lowest at pH 4.5 (Fig. 1A), which is in line with previous reports (Tsay *et al*., 1993; Fang *et al*., 2016). In the presence of NH_4_^+^, transcript levels dropped only at pH 5.5 and 6.5, while those at pH 4.5 even increased, indicating a pronounced pH dependency of the NH_4_^+^-dependent repression of *NRT1.1*. Surprisingly, maximum expression at pH 5.5 was attenuated in *aha2* and particularly in *qko* even in the absence of NH_4_^+^ (Fig. 1A), suggesting that the presence of AMTs or residual NH_4_^+^ uptake interfered with the transcriptional regulation of *NRT1.1*. Thus, the optimum *NRT1.1* expression at pH 5.5 was compromised not only by the presence of NH_4_^+^ but also by a lack of AHA2 or AMT activity.

**Fig. 2.**
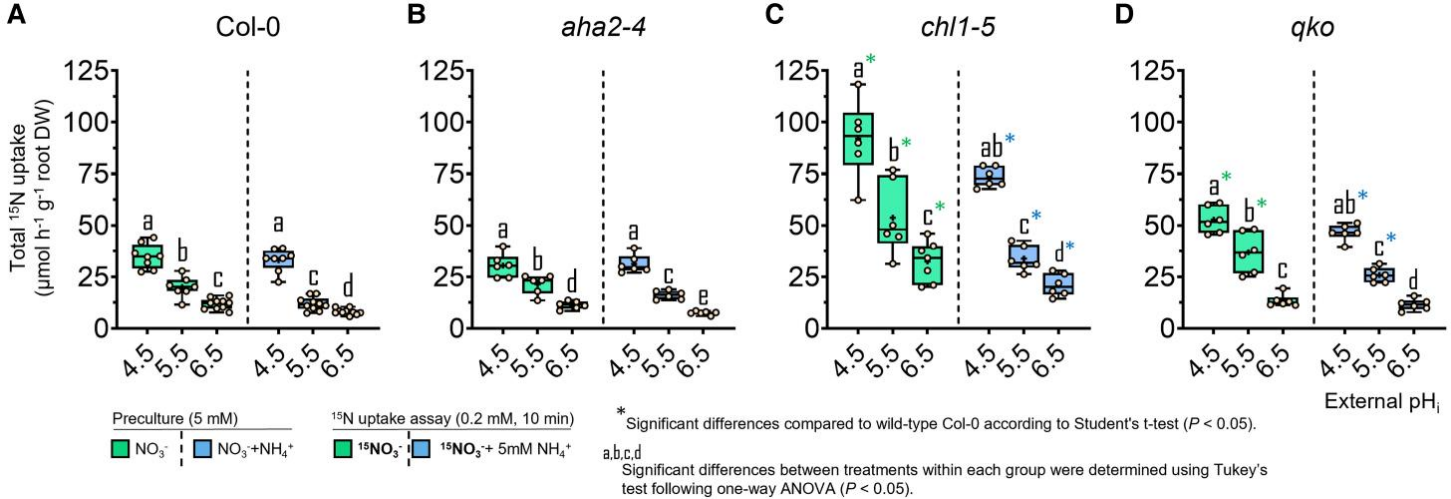
External H^+^ availability drives the major high-affinity NO_3_^−^ uptake rate in Arabidopsis. Influx of ^15^N-labelled NO_3_^-^ was measured in roots of 6-week-old wild-type (Col-0) plants (A) and the mutant lines *aha2-4* (B), *chl1-5* (C), and *qko* (D). Plants were precultured with either 2.5 mM Ca(NO_3_)_2_ or 1.25 mM (NH_4_)_2_SO_4_ + Ca(NO_3_)_2_ and subsequently exposed to an unbuffered nutrient solution containing 0.2 mM ^15^NO_3_^-^ for 10 min, supplied either alone or together with 5 mM NH_4_^+^, at external pH 4.5, 5.5, or 6.5. Box plots show the median (line) and mean (+), interquartile range (box), and minimum and maximum values (whiskers); *n* = 6–10. Different letters indicate significant differences among treatments within each genotype (one-way ANOVA followed by Tukey’s test, *P* < 0.05). Asterisks (*) denote significant differences relative to wild-type Col-0 within the same pH treatment (Student’s *t*-test, *P* < 0.05)..

As expected, the loss of *NRT1.1* expression in *chl1-5* was accompanied by a pronounced de-repression of *NRT2.1*, *NRT2.2*, *NRT2.4,* and *NRT2.5*, also when plants were pre-cultured in the presence of NH_4_^+^ (Fig. 1B–F). This explained in general the higher capacity for high-affinity NO_3_^−^ uptake in *chl1-5* than in the other lines (Muños *et al*., 2004; Krouk *et al*., 2006). As for *NRT1.1*, transcript levels of *NRT2* genes were lowest at pH 4.5, when NO_3_^−^ influx was highest (Fig. 2). Unlike *NRT1.1*, *NRT2* transcript levels were affected to a small extent by a lack of AHA2, whereas defective NH_4_^+^ transport in *qko* led to up-regulation of *NRT2* genes, in particular at pH 6.5. This up-regulation was largely sustained in roots pre-cultured under mixed N nutrition. Taken together, apart from the regulatory role of NRT1.1, external pH had a stronger impact on the regulation of *NRT2* genes than did the presence of NH_4_^+^.

**Fig. 3.**
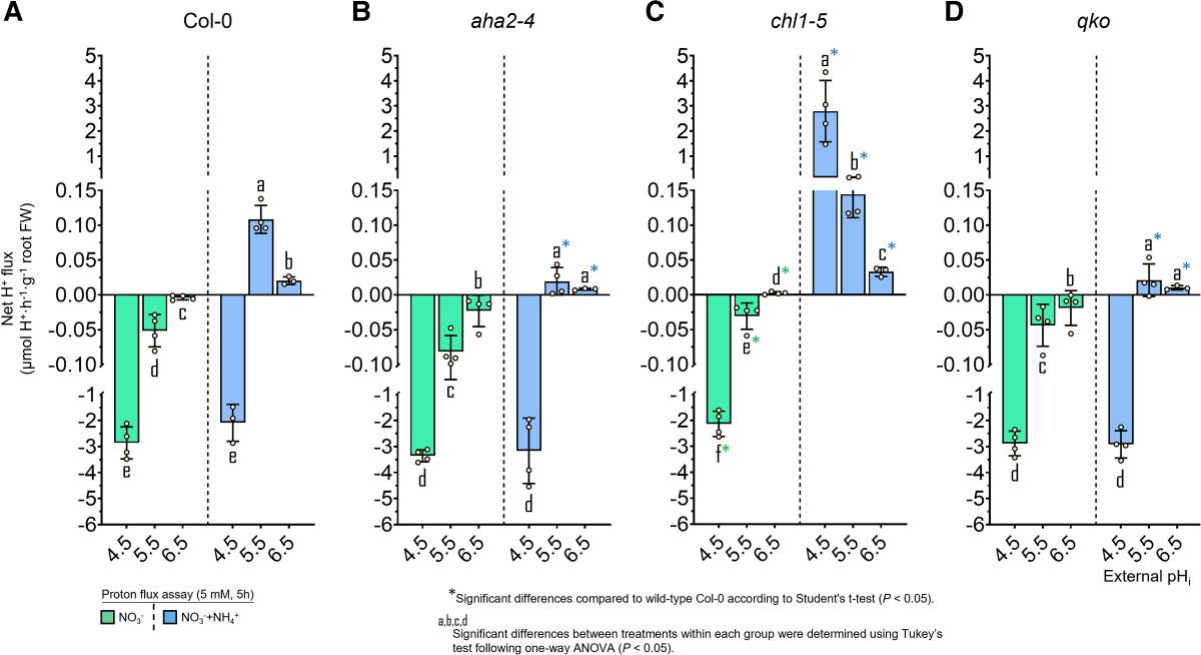
Net H^+^ fluxes under NO_3_^−^ nutrition reflect the NO_3_^−^ uptake capacities in Arabidopsis. Net H^+^ fluxes were measured in roots of 6-week-old wild-type (Col-0) plants (A) and the mutant lines *aha2-4* (B), *chl1-5* (C), and *qko* (D). Roots were immersed in an unbuffered nutrient solution adjusted to initial pH values of 4.5, 5.5, or 6.5 and supplied with either 2.5 mM Ca(NO_3_)_2_ or 1.25 mM (NH_4_)_2_SO_4_ + Ca(NO_3_)_2_. After 5 h, changes in H^+^ concentration were determined to calculate net H^+^ fluxes. Data represent means ± SE (*n* = 3–4). Different letters indicate statistically significant differences among treatments within each genotype (one-way ANOVA followed by Tukey’s test, *P* < 0.05). Asterisks (*) denote significant differences relative to wild-type Col-0 within the same pH treatment (Student’s *t*-test, *P* < 0.05)..

In wild-type plants, NO_3_^−^ influx was maximal at pH 4.5, accompanying elevated root N concentrations, and declined progressively with increasing pH (Fig. 2A; Supplementary Fig. S3A). Under co-supply of NH_4_^+^, NO_3_^−^ uptake decreased further only at pH 5.5 and 6.5, suggesting that suppression by concomitant NH_4_^+^ uptake had come into play. Notably, only at pH 5.5 did NO_3_^−^ uptake remain slightly higher in the *aha2-4* mutant (Fig. 2B), pointing to a weaker repression of NO_3_^−^ uptake as a consequence of weaker AHA2-dependent NH_4_^+^ uptake. Higher NO_3_^−^ influx at low pH was reflected by higher consumption of external H^+^ (Fig. 3). Only in the presence of NH_4_^+^ was the H^+^ balance positive at pH 5.5 (Fig. 3; Supplementary Fig. S4B), and to a lesser extent at pH 6.5 (Fig. 3), most probably as a consequence of lower NO_3_^−^ uptake and/or incipient NH_4_^+^ uptake. In support of the latter, H^+^ efflux in the presence of NH_4_^+^ decreases with loss of AHA2 (Fig. 3B). Nonetheless, AHA2 activity as well as the presence of NH_4_^+^ had an unexpectedly weak impact on (mainly NRT1.1-dependent) high-affinity NO_3_^−^ influx.

In the *chl1-5* mutant, high-affinity NO_3_^−^ influx was strongly enhanced, which must have resulted from de-repressed NRT2 activity (Ohkubo *et al*., 2021). NO_3_^−^ influx was at a >2-fold higher level than that in wild-type roots, and pH dependency as well as the suppressive impact of NH_4_^+^ co-supply on NO_3_^−^ influx were more pronounced. Despite the large NO_3_^−^ influx capacity in *chl1-5*, NH_4_^+^ co-supply changed the net H^+^ fluxes to positive (Fig. 3C), indicating concomitant NH_4_^+^ uptake. Hence, switching the dependence of NO_3_^−^ uptake from NRT1.1 to NRT2-type transporters in the presence of NH_4_^+^ decreased external H^+^ consumption and emphasized the dominant impact of NRT1.1 on the net H^+^ balance.

Next, we assessed the impact of concomitant NH_4_^+^ uptake on NO_3_^−^ influx, employing the *qko* mutant that exhibits only 5–10% residual high-affinity NH_4_^+^ uptake capacity (Yuan *et al*., 2013). As in the other lines, NO_3_^−^ influx decreased with increasing pH, and even more so in the presence of NH_4_^+^. However, *qko* showed a significantly higher NO_3_^−^ influx than Col-0, in particular at low pH. These genotype-dependent differences in short-term NO_3_^−^ influx were hardly reflected in the external H^+^ balance when considereded over a longer time scale (Fig. 3). Notably, the NO_3_^−^ uptake capacity in the *qko* mutant remained high in the presence of NH_4_^+^. This observation, combined with the fact that the pH-dependent decrease in uptake was comparable across all lines, strongly suggests that NH_4_^+^ itself, rather than its associated H^+^ fluxes, is responsible for suppressing NO_3_^−^ uptake. This allows the inhibitory effect of NH_4_^+^ to be decoupled from both the NH_4_^+^ uptake-associated H^+^ efflux (altered in *qko*) and the general H^+^ secretion (impaired in *aha2-4*) (Fig. 3B, D; Supplementary Fig. S4A, B). This assumption was further supported by the *chl1-5* mutant, in which NO_3_^−^ influx also decreased upon NH_4_^+^ co-provision at pH 5.5 and 6.5 despite a markedly higher net H^+^ efflux. Taken together, increasing NO_3_^−^ influx with decreasing pH was remarkably robust across all lines and only marginally affected by concomitant NH_4_^+^ uptake.

The remarkable phenotypic convergence between *qko* and *chl1-5* in their low pH-dependent high-affinity NO_3_^−^ uptake prompted us to test whether this regulation was dependent on the internal plant nutritional status. We therefore re-assessed the ^15^NO_3_^−^ uptake capacity in plants pre-cultured with 2.5 mM instead of 5 mM NO_3_^−^ (Supplementary Fig. S5). In Col-0 and *qko*, NO_3_^−^ influx increased significantly, reflecting a less saturated N status. Nonetheless, NO_3_^−^ influx in *qko* remained higher than in the wild type under the same pH conditions (pH 4.5 and 5.5). Despite the high NO_3_^−^ influx in *chl1-5*, the less saturating N pre-culture further increased influx, significantly at pH 5.5 and 6.5, pointing to a stronger contribution of NRT2-type transporters.

### Role of external H^+^ availability in gene expression of NH_4_^+^ transporters and NH_4_^+^ influx

**Fig. 4.**
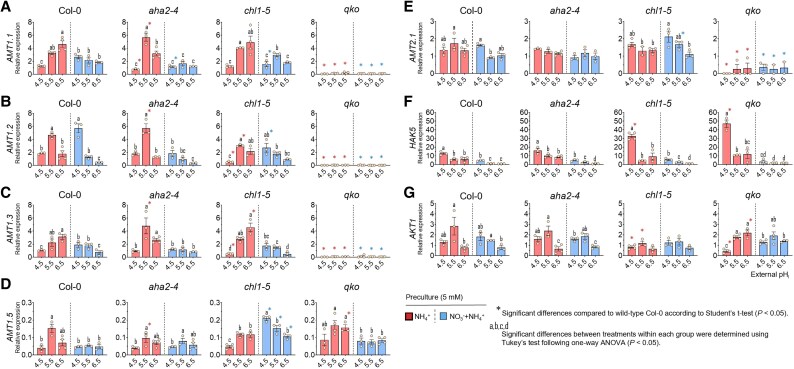
NO_3_^−^ co-supply moderates the transcriptional induction by higher pH of the major high-affinity NH_4_^+^ and K^+^ transporters, although it does not explain the pH-dependent pattern of NH_4_^+^ uptake among the different lines. Transcript levels of *AMT1.1* (A), *AMT1.2* (B), *AMT1.3* (C), *AMT1.5* (D), *AMT2.1* (E), *HAK5* (F), and *AKT1* (G) were quantified by real-time RT–PCR in roots of 6-week-old Arabidopsis wild-type (Col-0) and mutant lines (*aha2-4*, *chl1-5*, and *qko*). Plants were precultured with either NH_4_^+^ alone or a combined NO_3_^-^ + NH_4_^+^ nitrogen supply and exposed to external pH 4.5, 5.5, or 6.5. Transcript abundance was normalized to the constitutively expressed reference gene *UBQ10*. Data represent means ± SE (*n* = 3–4 biological replicates). Different letters indicate statistically significant differences among pH and nitrogen treatments within each genotype (one-way ANOVA followed by Tukey’s post hoc test, *P* < 0.05). Asterisks (*) denote significant differences relative to wild-type Col-0 under the same pH and nitrogen conditions (Student’s *t*-test, *P* < 0.05)..

When comparing steady-state expression levels of NH_4_^+^ transporter genes across all lines, it was most evident that cultivating plants at pH 4.5 strongly repressed the transcript levels of all *AMT1* genes under sole NH_4_^+^ nutrition (Fig. 4A–D). Under co-cultivation with NO_3_^−^, this low pH-dependent repression was alleviated, with the strongest effect observed on *AMT1.2*, which demonstrated a 3-fold increase in transcript levels. In the *aha2* and *chl1-5* mutants, the low pH-dependent repression of *AMT1* transcript levels was similar to that in wild-type plants; however, the relief in the presence of NO_3_^−^ was weaker. In *chl1-5*, an exception was *AMT1.5*, whose mRNA levels increased in the presence of NO_3_^−^. At pH 5.5 or 6.5, the relieving effect of NO_3_^−^ became generally weaker. Transcript levels of *AMT2.1*, which significantly contributes to NH_4_^+^ uptake under high NH_4_^+^ supply (Giehl *et al*., 2017), remained unaffected by either pH or the mutant background.

**Fig. 5.**
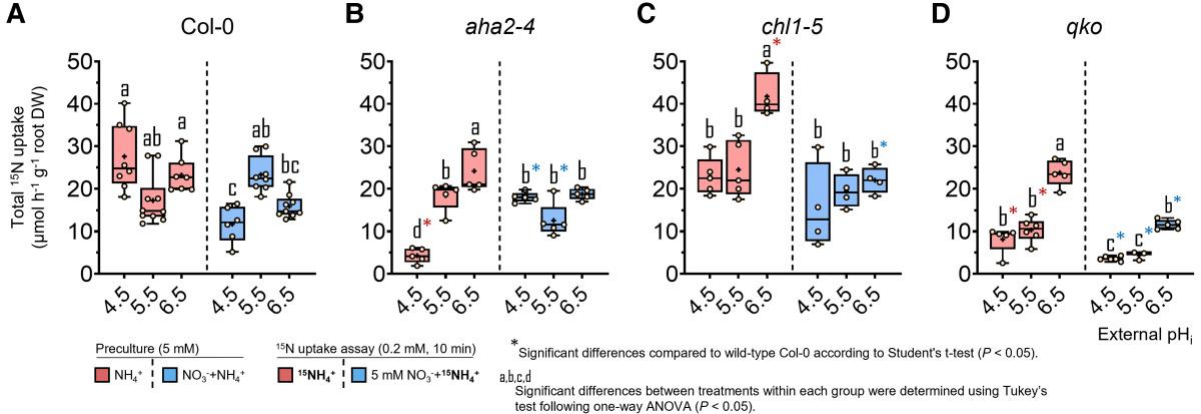
AHA2 at low pH, and NRT1.1 and AMTs at high pH, play a crucial role in NH_4_^+^ uptake. Influx of ^15^N-labelled NH_4_^+^ was measured in roots of 6-week-old Arabidopsis wild-type (Col-0) (A) and the mutant lines *aha2-4* (B), *chl1-5* (C), and *qko* (D). Plants were precultured with 2.5 mM (NH_4_)_2_SO_4_ or an equimolar mixed nitrogen supply (1.25 mM (NH_4_)_2_SO_4_ + Ca(NO_3_)_2_) and subsequently exposed for 10 min to an unbuffered nutrient solution containing 0.2 mM ^15^NH₄⁺ at external pH 4.5, 5.5, or 6.5. Box plots show the median (line), mean (+), interquartile range (box), and minimum and maximum values (whiskers); *n* = 5–8 biological replicates. Different letters indicate statistically significant differences among pH and nitrogen treatments within each genotype (one-way ANOVA followed by Tukey’s post hoc test, *P* < 0.05). Asterisks (*) denote significant differences relative to the wild-type Col-0 under the same pH and nitrogen conditions (Student’s *t*-test, *P* < 0.05)..

Next, we investigated the impact of external pH on NH_4_^+^ influx and on the balanced uptake of both N forms from the perspective of NH_4_^+^. Unlike NO_3_^−^, NH_4_^+^ influx did not follow a uniform pH-dependent pattern across all lines, but rather exhibited mutant-specific responses. Under exclusive NH_4_^+^ supply, Col-0 showed no significant differences in NH_4_^+^ influx across the external pH levels (Fig. 5A), supporting the notion that NH_4_^+^ uptake in Arabidopsis remains largely unaffected by external pH in this range.

**Fig. 6.**
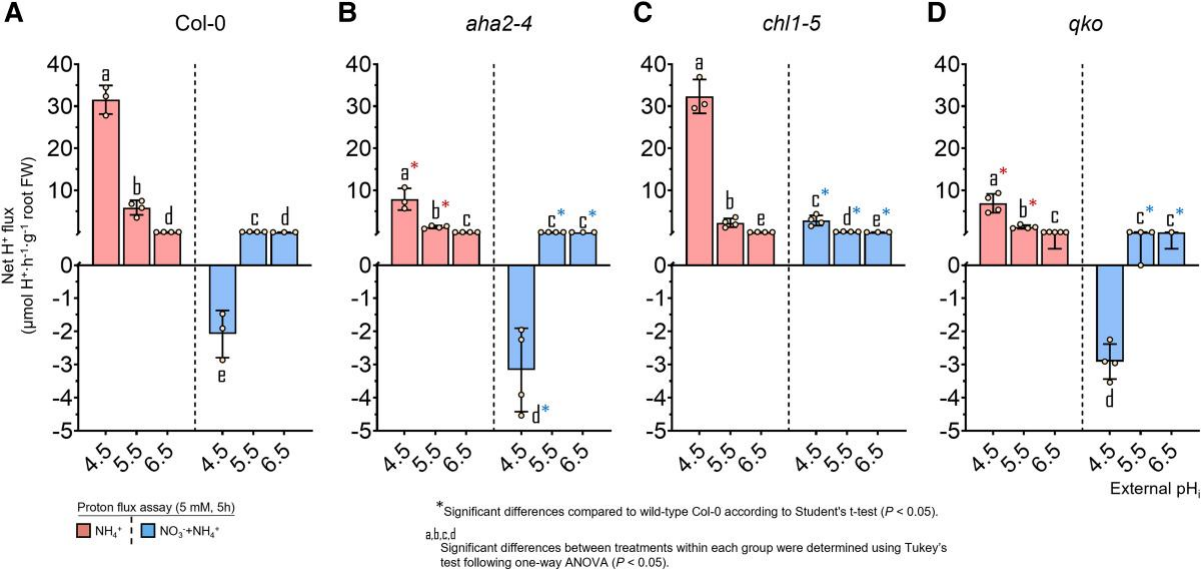
The net H^+^ fluxes under NH_4_^+^ nutrition do not reflect NH_4_^+^ uptake capacities in Arabidopsis. Net H^+^ fluxes were measured in roots of 6-week-old wild-type plants (Col-0) (A) and the mutant lines *aha2-4* (B), *chl1-5* (C), and *qko* (D). Roots were immersed in an unbuffered nutrient solution adjusted to an initial external pH of 4.5, 5.5, or 6.5 and supplied with either 2.5 mM (NH_4_)_2_SO_4_ (NH_4_^+^ alone) or a mixed nitrogen source consisting of 1.25 mM (NH_4_)_2_SO_4_ plus 1.25 mM Ca(NO_3_)_2_ (NO_3_^-^ + NH_4_^+^). Net H^+^ fluxes were determined after 5 h of treatment. Data represent the mean ±SE (*n* = 3–5). Different letters indicate statistically significant differences among Arabidopsis lines (one-way ANOVA; Tukey’s test, *P*<0.05). Asterisks (*) denote significant differences relative to the wild type (Col-0) within the same pH treatment (Student’s *t*-test, *P* < 0.05). Note that the N-mixed treatment (as shown in Fig. 2) are presented again here to allow direct comparison with the sole NH_4_^+^ treatment.

As expected, NH_4_^+^ influx declined in the *qko* mutant, albeit only at pH 4.5 and 5.5, while it recovered at pH 6.5, indicating the involvement of uptake pathways other than AMTs. In *aha2*, NH_4_^+^ uptake also decreased drastically, but only at pH 4.5 (Fig. 5B; Supplementary Fig. S6), highlighting the critical role of AHA2 in H^+^ secretion for efficient NH_4_^+^ uptake at low pH. Indeed, the loss of either AHA2 or the AMTs resulted in a nearly identical decrease in net H^+^ efflux at low pH. In contrast, NH_4_^+^ influx in *chl1-5* was similar to that in the wild type at low pH but increased strongly at pH 6.5 (Fig. 5C), theoretically implying the presence of an additional, NRT1.1-repressible NH_4_^+^ uptake pathway. This observed enhancement of NH_4_^+^ uptake at pH 6.5 was not linked to changes in the external H^+^ balance (Fig. 6).

To verify the impact of the N nutritional status on the regulation of NH_4_^+^ influx and its dependence on external pH, the NH_4_^+^ uptake assay was repeated with a lower NH_4_^+^ concentration of 2.5 mM during hydroponic culture. In general, NH_4_^+^ uptake rates increased by 2- to 3-fold, most probably as a consequence of a less saturated N status (Supplementary Fig. S6). The significantly higher NH_4_^+^ uptake capacity at pH 6.5 was confirmed in all three mutants.

Generally, NH_4_^+^ influx decreased in the presence of NO_3_^−^. In Col-0, the repression of NH_4_^+^ influx by external NO_3_^−^ was most pronounced at pH 4.5, namely under conditions where NO_3_^−^ influx was highest. This observation also held true for *qko*, in which NO_3_^−^ repressed NH_4_^+^ influx at all pH values, implying that NO_3_^−^ negatively interferes with NH_4_^+^ uptake irrespective of an AMT-dependent uptake pathway. In *chl1-5*, the elevated NH_4_^+^ influx at pH 6.5 decreased in the presence of NO_3_^−^. The only exception to NO_3_^−^ repression was observed in *aha2*, where the co-supply of NO_3_^−^ stimulated NH_4_^+^ influx at pH 4.5. While the loss of AHA2 activity was also reflected in a more negative H^+^ balance (Fig. 6B), co-supply of NO_3_^−^ appeared to compensate for the strong dependency of NH_4_^+^ influx on AHA2.

**Fig. 7.**
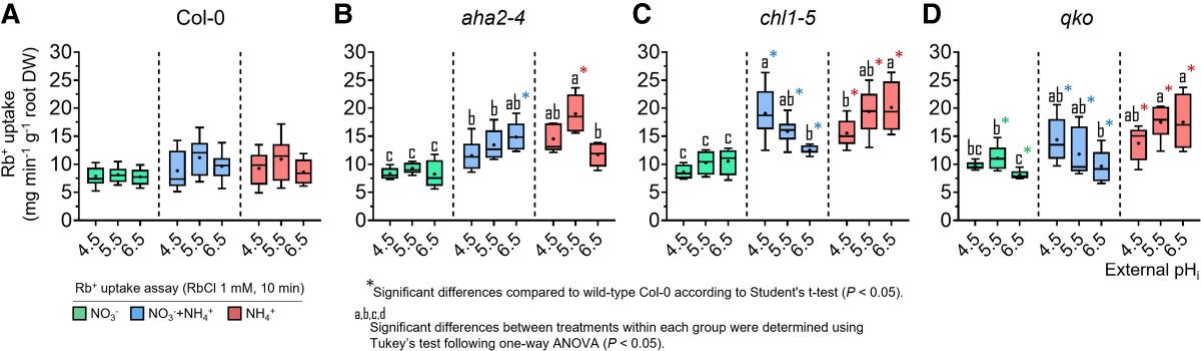
NH_4_^+^ nutrition further stimulates the Rb^+^ (∼K^+^) influx in *chl1-5* and *qko* mutants. Significant differences in Rb^+^ influx uptake are illustrated among the Arabidopsis lines (A, wild-type Col-0; B, *aha2-4*; C, *chl1-5*; D, *qko*) based on the treatments for each external pH. Box plots show the median (line) and mean (+), interquartile range (box), and minimum and maximum values (whiskers), *n*=6–10. Different letters indicate statistically significant differences among Arabidopsis lines (one-way ANOVA; Tukey’s test, *P*<0.05). Asterisks (*) denote significant differences relative to the wild type (Col-0) within the same pH treatment (Student’s *t*-test, *P*<0.05).

Given that non-specific cation channels have been proposed as alternative low-affinity pathways for NH_4_^+^ uptake and that Rb^+^ can be used as a substrate to examine channel-mediated cation transport (Britto and Kronzucker, 2006; ten Hoopen *et al*., 2010; Coskun and Kronzucker, 2013), Rb^+^ influx was measured and used here as a readout for the potential contribution of non-specific cation channels to NH_4_^+^ uptake (Fig. 7A). Under sole NO_3_^−^ nutrition, external pH had no considerable impact on Rb^+^ influx in any of the lines. Contrary to the expectation that NH_4_^+^ will inhibit Rb^+^ uptake, Rb^+^ influx remained unaffected in Col-0 and was even higher in the mutant lines. Interestingly, this NH_4_^+^-dependent elevation of Rb^+^ influx was largely sustained when plants were grown on NH_4_^+^ and NO_3_^−^. Relative to Col-0, Rb^+^ uptake in *chl1-5* and *qko* increased at pH 6.5 under sole NH_4_^+^ nutrition (Fig. 7C, D), mirroring the same trend observed for NH_4_^+^ influx. However, this difference was reversed by NO_3_^−^ co-supply, suggesting that their higher NO_3_^−^ uptake activity at low pH was associated with a greater demand for cations. In *aha2*, the positive effect of NH_4_^+^ on net Rb^+^ influx showed a different pH dependency. Upon analyzing the expression of the two major K^+^ transporter genes, *AKT1* and *HAK5*, whose products are capable of transporting Rb^+^ and potentially NH_4_^+^ (Rubio *et al*., 2008; ten Hoopen *et al*., 2010; Maierhofer *et al*., 2024), *HAK5* displayed a marked repression by NO_3_^−^ as well as by increasing pH across all lines. Notably, a distinct expression peak of *HAK5* was observed at pH 4.5 under sole NH_4_^+^ nutrition in *qko* and *chl1-5*. Transcript levels of *AKT1* were only slightly and not consistently affected by pH and N forms across the different lines.

## Discussion

As N uptake can constitute up to 70% of the total mineral element uptake (Marschner, 2012), the ratio of NH_4_^+^ to NO_3_^−^ uptake, together with the availability of H^+^, plays a critical role in maintaining a balanced uptake of cations and anions. To better understand the physiological processes governing the balanced uptake of both N forms, we measured the uptake rates of each form, in the absence and presence of the other, at different external pH values.

First, we examined the effect of external pH on NO_3_^−^ uptake, hypothesizing that a lower pH will enhance NO_3_^−^ uptake due to the H^+^ required for the 2H^+^/NO_3_^−^ co-transport by NRT1- and NRT2-type transporters (Tsay *et al*., 1993; Wirth *et al*., 2007). Indeed, in all examined lines, NO_3_^−^ influx was highest at pH 4.5 and decreased progressively with increasing pH (Fig. 2), confirming that external H^+^ availability is a major driving force for NO_3_^−^ uptake. This held true for both NRT1- and NRT2-mediated transport, since in *chl1-5* the de-repression of NRT2-dependent transport increased NO_3_^−^ influx by ∼3-fold, while still showing the same stimulation by high external H^+^ availability (Fig. 2A, C). Notably, transcript levels of *NRT1.1* and *NRT2* genes were consistently lowest at pH 4.5, even in *chl1-5* (Fig. 1E, F). This might represent a compensatory response to the high H^+^-motive force driving NO_3_^−^ uptake and a lower need for high NRT transporter abundance. To further evaluate whether changes in the H^+^-motive force directly constrain NO_3_^−^ uptake, we examined the *aha2* mutant (Haruta *et al*., 2010). Although AHA2 appears to be more relevant under alkaline conditions (Haruta *et al*., 2010; Jain and Schmidt, 2024), it is still expected to acidify the apoplast in order to drive H^+^-coupled NO_3_^−^ influx. However, NO_3_^−^ influx in *aha2* was the same as in Col-0 at pH 4.5, indicating that overall external H^+^ availability rather than the electrochemical gradient drives NO_3_^−^ uptake.

Intriguingly, the elevated NO_3_^−^ uptake in *qko* was not associated with transcriptional up-regulation of *NRT2* genes as seen in *chl1-5* and occurred even in the absence of external NH_4_^+^. This suggests a signaling role for AMTs beyond their function as NH_4_^+^ transporters, reminiscent of the NO_3_^−^-independent signaling role described for NRT1.1 in the regulation of NH_4_^+^ uptake (Hachiya and Noguchi, 2011; Jian *et al*., 2018; Hachiya *et al*., 2024). Further research is needed to determine whether AMTs contribute to the regulation of NO_3_^−^ uptake in a manner analogous to the recently proposed NRT1.1–QSK1–AHA2 module, particularly under varying N ratios and concentrations (Zhu *et al*., 2024).

On the other hand, NH_4_^+^ uptake did not benefit from low external pH. Sole NH_4_^+^ nutrition under acidic conditions at pH 4.5 is compromising the establishment of the electrochemical gradient across the PM (Haruta *et al*., 2010; Marschner, 2012; Yuan *et al*., 2017). This may reduce the driving force required for efficient NH_4_^+^ uptake, as AHA2-dependent H^+^ efflux is essential to sustain NH_4_^+^ influx under such conditions (Fig. 5B). By contrast, the *chl1-5* mutant did not display the NH_4_^+^-tolerant phenotype (Supplementary Fig. S1) previously reported by Hachiya and Noguchi (2011) and Hachiya *et al*. (2024). The potential influence of the NH_4_^+^ salt counter-anion was considered as a cause of this discrepancy. Since NRT1.1 has been proposed to facilitate chloride (Cl^−^) uptake under certain conditions, and Cl^−^ is known to exacerbate NH_4_^+^ toxicity (Liu *et al*., 2020), it is hypothesized that the tolerance of *chl1-5* described by Hachiya *et al*. might partially reflect reduced sensitivity to the co-supply of KCl alongside (NH_4_)_2_SO_4_. Indeed, other studies utilizing only (NH_4_)_2_SO_4_ or ammonium succinate failed to detect a significant growth advantage in *chl1-5* with respect to Col-0 (Liu *et al*., 2020, 2022). However, our discrepancy with the NH_4_^+^-tolerant phenotype of *chl1-5* observed in Jian *et al*. (2018) and Xiao *et al*. (2022), where (NH_4_)_2_SO_4_ was used, therefore requires another explanation.

Second, we evaluated the reciprocal influence of NO_3_^−^ and NH_4_^+^ on the uptake capacity of the other N form. We hypothesized that the H^+^ fluxes linked to AMT- and NRT-dependent transport will mutually facilitate the NO_3_^−^ and NH_4_^+^ influx, thereby maintaining the NO_3_^−^–NH_4_^+^ uptake balance. However, at pH 4.5, NO_3_^−^ influx was not affected by the presence of NH_4_^+^ (Fig. 2). Only at pH ≥5.5 did NH_4_^+^ consistently repress NO_3_^−^ uptake across all lines, coinciding with lower transcript levels of *NRT2* genes at higher pH (Fig. 1E, F). Although previous studies demonstrated that *NRT2.1* is the primary target of NH_4_^+^-dependent inhibition of high-affinity NO_3_^−^ uptake in Arabidopsis (Cerezo *et al*., 2001; Muños *et al*., 2004; Wirth *et al*., 2007), our study highlights that this inhibition does not occur under acidic conditions. Moreover, the repression of NO_3_^−^ uptake by NH_4_^+^ was still observed in the *qko* mutant, despite its weak NH_4_^+^ uptake activity, pointing to NH_4_^+^ uptake pathways other than AMTs, that can still trigger the repression of NO_3_^−^ uptake. Therefore, NH_4_^+^-dependent repression of NO_3_^−^ uptake gains importance only when the driving force of high H^+^ availability for NO_3_^−^ co-transport is declining (i.e. at pH >4.5).

On the other hand, at pH 4.5, NH_4_^+^ influx was negatively affected by the presence of NO_3_^−^ in the wild type but not in *chl1-5* (Fig. 5A, C). This indicates that enhanced NO_3_^−^ uptake under acidic conditions, mediated by increased H^+^ co-transport (Figs 2, 3), perturbs the PM electrochemical gradient. The massive H^+^ entry accompanying NO_3_^−^ may locally deplete apoplastic H^+^, effectively consuming external H^+^ and depolarizing the membrane potential to such an extent that NH_4_^+^ influx decreases. Under these conditions, NRT1.1 appears to be most responsible for this strong H^+^-coupled NO_3_^−^ influx, as the NO_3_^−^-dependent inhibition of NH_4_^+^ uptake was absent in *chl1-5*. Likewise, at pH 6.5, the H^+^-motive force is diminished not only by the intrinsically lower H^+^ concentration but also by active depletion of H^+^ via NO_3_^−^ co-transport, further weakening the driving force across the PM. This could explain the lower NH_4_^+^ influx observed across all genotypes under mixed N conditions. Together, these results suggest that NRT1.1-dependent H^+^/NO_3_^−^ co-transport, in particular, competes for external H^+^ at low pH, indirectly repressing NH_4_^+^ influx and also Rb^+^ uptake (Fig. 7C) by compromising the electrochemical gradient.

Arabidopsis appears to prioritize maintaining a stable total N content over a strict NO_3_^−^–NH_4_^+^ balance. Accordingly, the disruption of NO_3_^−^ or NH_4_^+^ transport in *chl1-5* or *qko*, respectively, reflected by lower root N content, tends to be compensated by increased uptake of NH_4_^+^ and NO_3_^−^ at higher pH (Supplementary Fig. S3), although NO_3_^−^ acquisition by NRT1.1 remains crucial for sustaining the stronger NO_3_^−^-dependent shoot growth (Supplementary Fig. S2A). This underscores a key asymmetry in the regulation of NO_3_^−^ and NH_4_^+^ balance: while NO_3_^−^ uptake capacity is robustly fueled by the external H^+^ availability and by the activity of functional NRT1.1, NH_4_^+^ uptake capacity depends heavily on AHA2 to maintain the PM potential and stabilize it against the unfavorable H^+^ gradient as a consequence of sole NH_4_^+^ and low pH conditions. When compared with near-neutral conditions, it becomes evident that the regulation of balanced N uptake shifts from an NRT1.1-dependent H^+^ driven mechanism to a transcriptionally mediated one: NH_4_^+^ prevents the induction of *NRT2* genes, and NO_3_^−^ that of *AMT1* genes. This dynamic adjustment underscores the flexibility and robustness of the NRTs and AMTs in maintaining N homeostasis across variable environmental conditions. It is conceivable that under acidic natural conditions, the reduced availability of NO_3_^−^ may be counterbalanced by the elevated H^+^ gradient. This, in turn, leads to an increase in rhizosphere pH, helping to mitigate further low-pH stress within the plant. As pH increases, higher NO_3_^−^ availability and the transcriptional induction of the high-affinity transporters compensate for the lower H^+^-dependent NO_3_^−^ uptake, reducing the need for additional AHA2 activity and energy. This effect is amplified in the absence of NRT1.1, where impaired NO_3_^−^ uptake leads to de-repression of NRT2 expression and activity (Fig. 3B–D, G), as well as reduced H^+^ consumption, which is partially compensated by increased NH_4_^+^ acquisition. Interestingly, a similar response is observed in the absence of AMTs, even when no external NH_4_^+^ is present. This parallel behavior between *chl1-5* and *qko* mutants is also evident at higher pH during NH_4_^+^ influx in the absence of NO_3_^−^, where roots presumably acquire more NH_4_^+^ either through increased AMT activity or via alternative cation channels to ensure total N acquisition.

**Fig. 8.**
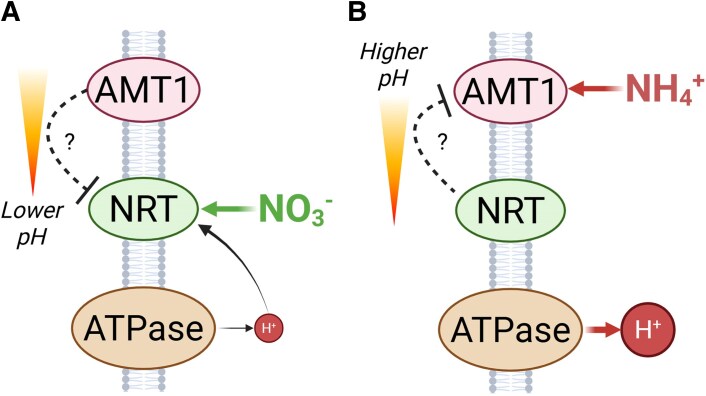
Proposed model illustrating the putative indirect interplay between NRTs and AMTs during high-affinity NO_3_^−^ and NH_4_^+^ uptake. (A) High-affinity NO_3_^−^ uptake mediated by NRTs and co-transported with H^+^ increases as external pH declines. AMTs appear to indirectly exert a negative effect on high-affinity NO_3_^−^ transporters to further restrict NO_3_^−^ uptake at low pH, and independently of NH_4_^+^ co-supply. (B) High-affinity NH_4_^+^ uptake mediated by AMTs contributes to external medium acidification, partially dependent on H^+^-ATPase AHA2, with stronger effects at lower pH. At higher pH, by contrast, NRT1.1 appears to indirectly exert a negative influence on AMTs, limiting NH_4_^+^ uptake. The contribution of functional NO_3_^−^ or NH_4_^+^ uptake capacities modulates the magnitude of these responses rather than determining overall N uptake patterns.

By employing a controlled hydroponic system to decouple N nutrition from external pH, a functional interconnection between NRTs and AMTs is revealed. It is shown that the absence of one N form enhances the acquisition of the alternative N form in a pH-dependent manner, a mechanism contributing to the maintenance of the total plant N status (Fig. 8). An exciting avenue is thus opened up for future research into the putative NO_3_^−^- and NH_4_^+^-independent signaling pathways that orchestrate this crosstalk.

## Supplementary Material

erag007_Supplementary_Data

## Data Availability

All data generated or analyzed during this study are included in this published article and its supplementary data files.

## Supplementary data

The following supplementary data are available at *JXB* online.

Table S1. Raw Ct values for candidate housekeeping genes used in RT–qPCR analysis.

Table S2. Gene-specific primers used for qPCR in Arabidopsis roots.

Fig. S1. NO_3_^−^ nutrition sustains significant plant growth compared with NH_4_^+^ as the sole N form, which exacerbates overall growth arrest under low pH conditions in root length, shoot FW, and root FW.

Fig. S2. Low external pH leads to further cytosolic acidification in root and shoot cells when NH_4_^+^ is the sole N form, whereas NO_3_^−^ nutrition acts as a buffer that mitigates this acidification.

Fig. S3. The higher NO_3_^−^ uptake at low pH enhances total N content in an NRT1.1-dependent manner, whereas NH_4_^+^ uptake under strict NH_4_^+^ conditions relies on AMT and AHA2.

Fig. S4. Changes in the external pH relative to the initial buffered pH under the different N regimes.

Fig. S5. A direct comparison between two independent experiments underscores the reproducibility of NO_3_^−^ uptake enhancement under acidic conditions, and points to AMTs as potential upstream mediators of this process.

Fig. S6. A direct comparison between two independent experiments underscores the reproducible involvement of AHA2 in NH_4_^+^ uptake at pH 4.5, and suggests the existence of alternative transport systems that compensate for the loss of AMT function in the *qko* mutant at pH 6.5.
