# Supplementary material for: Reciprocal co-regulation of nitrate and ammonium transporters is modulated by external pH in Arabidopsis
Source: J Exp Bot. 2026 Jan 13;77(8):2456–67. doi: 10.1093/jxb/erag007 (PMC13080360; doi:10.1093/jxb/erag007)
Supplement: erag007_Supplementary_Data [file erag007_supplementary_data.zip › jexbot316315-file001.pdf]

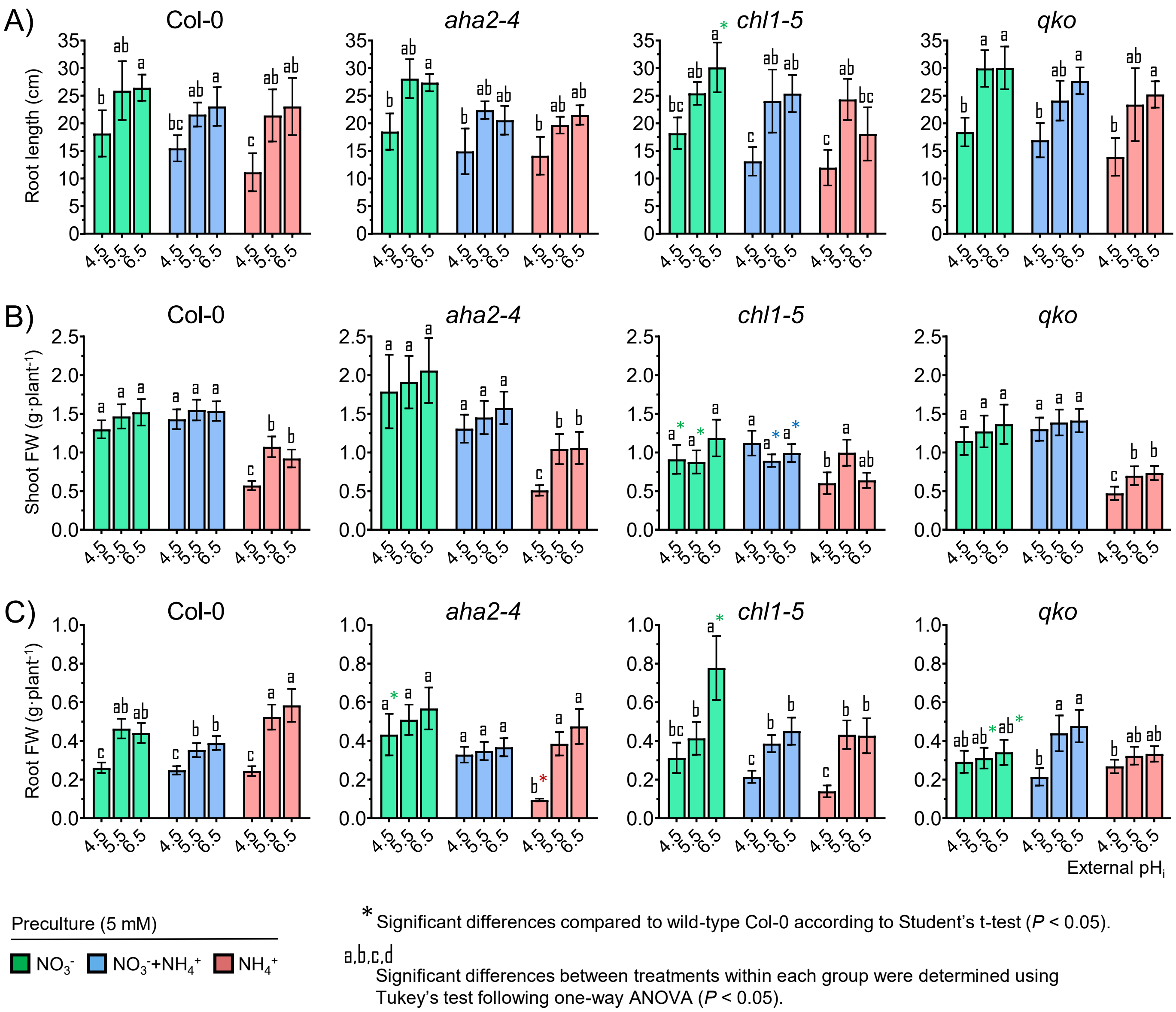

Fig. S1. NO_3^- nutrition sustains significant plant growth compared to NH_4^+ as the sole N form, which exacerbates overall growth arrest under low pH conditions in A) root length; **B)** shoot fresh weight, and **C)** root fresh weight. Hydroponically-grown plants were cultured for six weeks in buffered nutrient solution containing 2.5 mM $Ca(NO_3)_2$, 2.5 mM $(NH_4)_2SO_4$, or 1.25 mM of each salt at pH of 4.5, 5.5, or 6.5. Data represent mean ± SD (n = 12-16). Different letters indicate statistically significant differences among Arabidopsis lines (one-way ANOVA; Tukey's test, $P < 0.05$), while the asterisk symbol (*) denotes significant differences compared to the same treatment in the wild-type Col-0 (t Student's, $P < 0.05$).

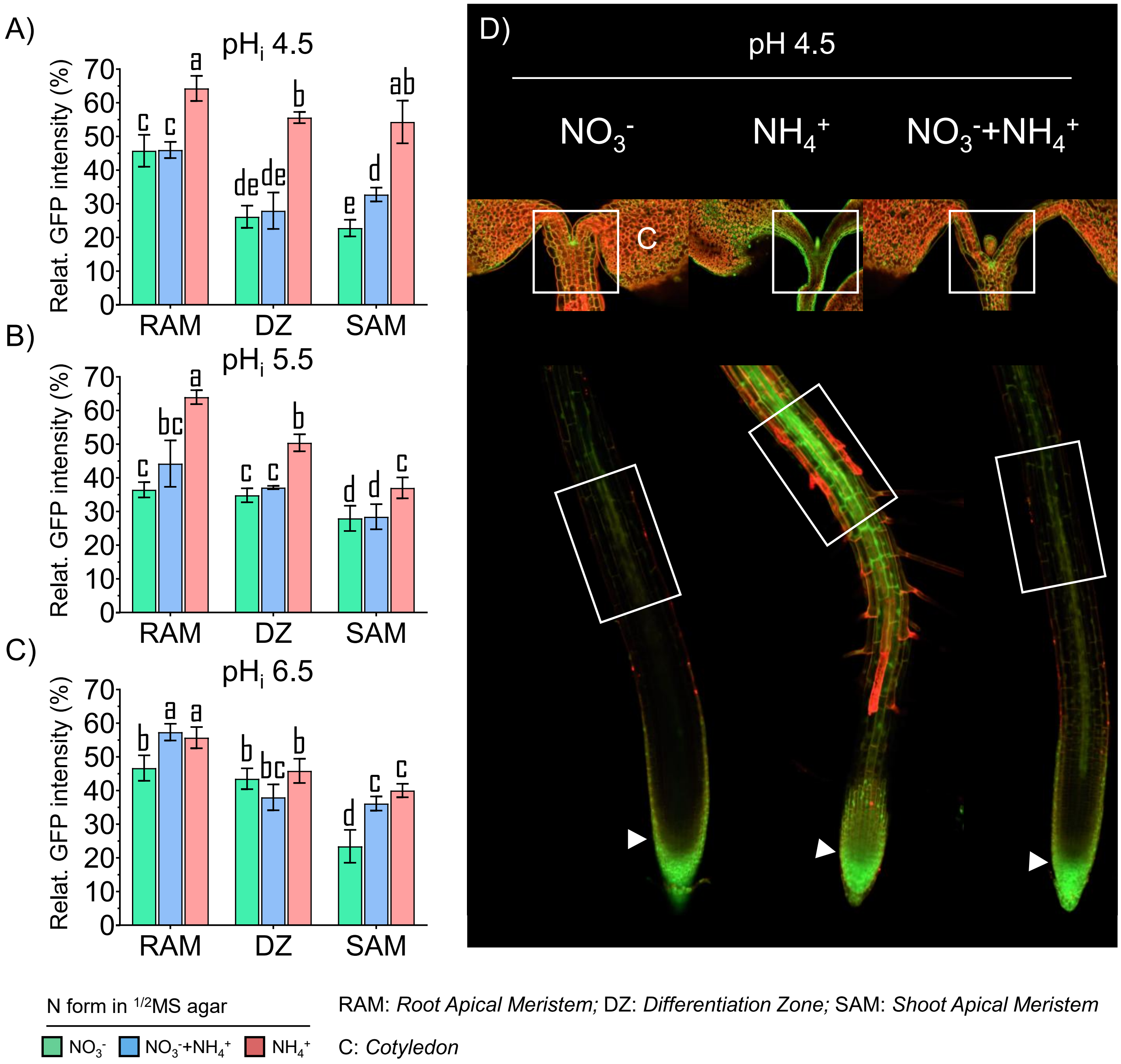

Fig. S2. Low external pH leads to further cytosolic acidification in root and shoot cells when NH_4^+ is the sole N form, whereas NO_3^- nutrition acts as a buffer that mitigates this acidification. A–C) Relative GFP signal intensity of the cytosolic pH sensor PtGFP in 8-day-old Arabidopsis seedlings (Col-0), measured in root (root apical meristem, RAM, and differentiation zone, DZ) and shoot (shoot apical meristem, SAM), after exposure to an initial pH (pH_i) of **A)** 4.5, **B)** 5.5, and **C)** 6.5. Data represent mean ± SE (n = 5). Different letters indicate statistically significant differences among N treatments at a total concentration of 10 mM (one-way ANOVA; Tukey's test, $P < 0.05$). Higher values indicate stronger cytosolic acidification. **D)** Representative image of cytosolic acidification in Arabidopsis seedlings at pH 4.5 under the three N regimes. Note how the presence of NO_3^- counteracts the acidification induced by NH_4^+. From top to bottom within the inset: SAM (*c* indicates cotyledon), DZ, and arrow pointing to the RAM. Greener colour indicates stronger cytosolic acidification. The red colour shows propidium iodide (PI) staining, which outlines the cell boundarie. Histological staining and fluorescent imaging were performed 3 days after treatments. The agar medium was buffered with 5 mM MES+Tris at the indicated pHs.

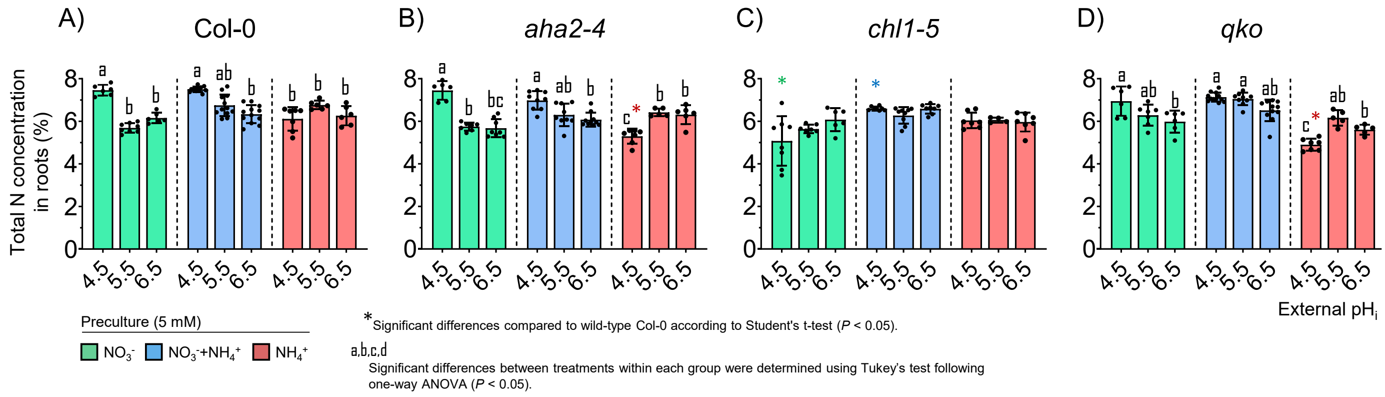

Fig. S3. The higher NO_3^- uptake at low pH enhances total N content in an NRT1.1-dependent manner, whereas NH_4^+ uptake under strict NH_4^+ conditions relies on AMT and AHA2. Hydroponically-grown plants were cultured for six weeks in buffered nutrient solution containing 2.5 mM $Ca(NO_3)_2$, 2.5 mM $(NH_4)_2SO_4$, or 1.25 mM of each salt at pH of 4.5, 5.5, or 6.5. Data represent mean ± SD (n = 6-10). Different letters indicate statistically significant differences among Arabidopsis lines (one-way ANOVA; Tukey's test, P < 0.05), while the asterisk symbol (*) denotes significant differences compared to the same treatment in the wild-type Col-0 (t Student's, P < 0.05).

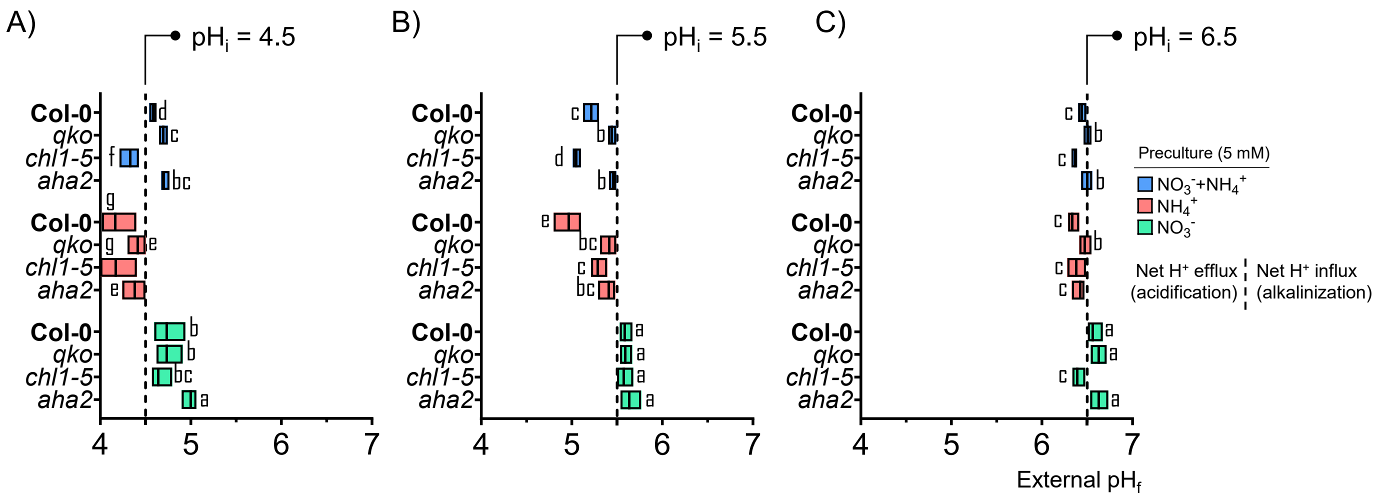

Fig. S4. Changes in the external pH relative to the initial buffered pH under the different N regimes. The initial pH (pH$_i$) was adjusted with 5 mM MES and drops of NaOH (5 M) to precisely reach **A)** 4.5, **B)** 5.5, or **C)** 6.5. Final pH (pH$_f$) was measured in each 5 l hydroponic pot per line between weeks 4 and 6 of growth, once the three N regimes were implemented and root biomass was sufficient to affect the external pH. Bars indicate the range of final pH values (horizontal line within the bar shows the mean, n = 6). Final pH values below the initial pH indicate acidification (net H^+ efflux), whereas values above the initial pH indicate alkalinization (net H^+ influx). Floating bars represent mean ± SE (n = 3). Different letters denote significant differences among the Arabidopsis lines (one-way ANOVA followed by Tukey's test, P < 0.05).

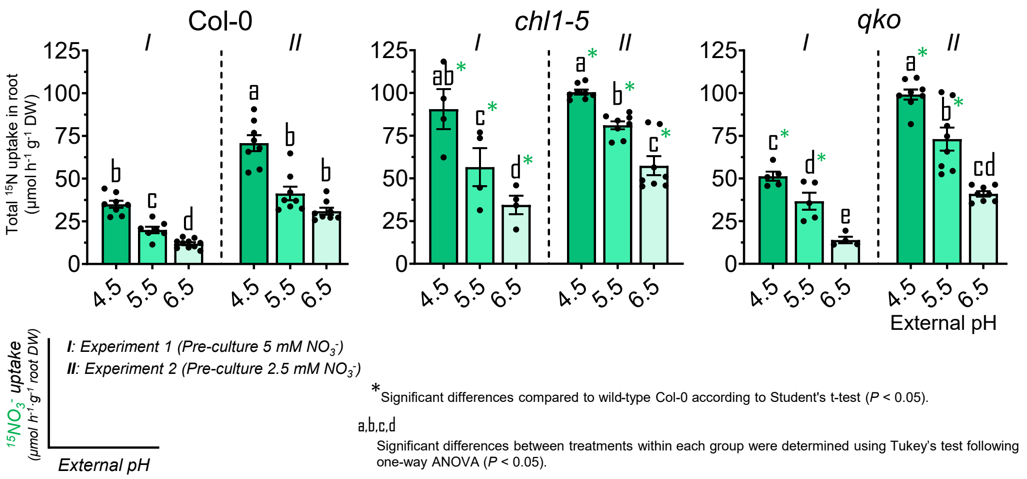

Fig. S5. A direct comparison between two independent experiments (I and II) underscores the reproducibility of the NO_3^- uptake enhancement under acidic conditions, and points to AMTs as potential upstream mediators of this process. Influx of ^{15}N-labelled NO_3^- was measured in roots of 6-week-old plants pre-cultured with either 2.5 mM $Ca(NO_3)_2$ (Experiment I) or 1.25 mM (Experiment II), and subsequently exposed for 10 minutes to an unbuffered nutrient solution containing 0.1 mM $Ca(NO_3)_2$. Data represent mean ± SE (n = 5-8). Different letters indicate statistically significant differences among genotypes (one-way ANOVA, Tukey's HSD test, $P < 0.05$). Asterisks (*) denote significant differences relative to the wild-type (Col-0) within the same pH treatment (Student's t-test, $P < 0.05$).

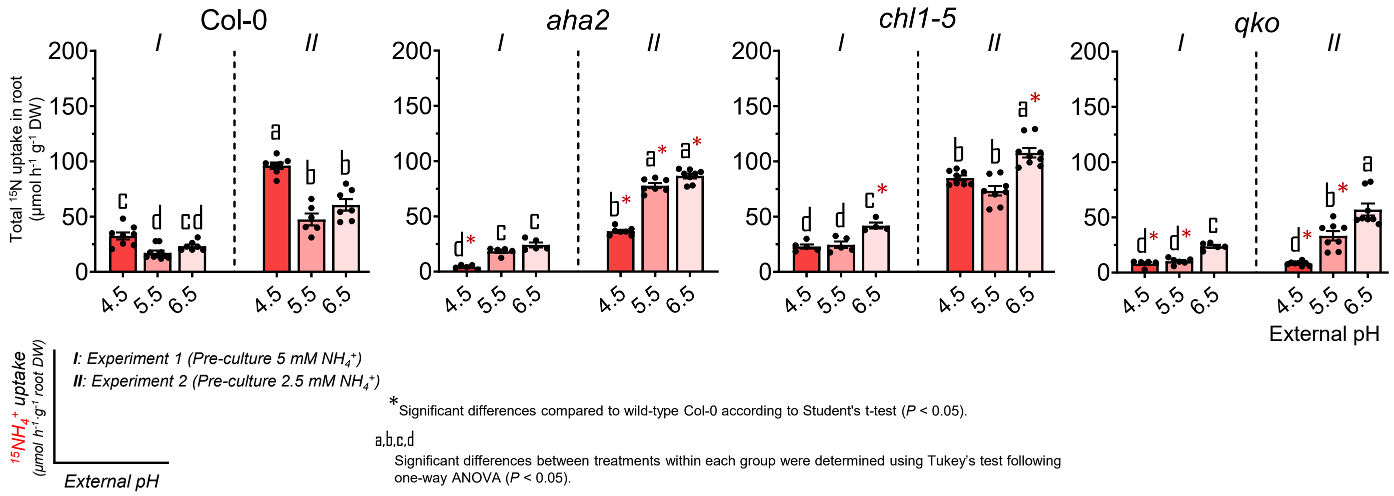

Fig. S6. A direct comparison between two independent experiments (I and II) underscores the reproducible involvement of AHA2 in NH_4^+ uptake at pH 4.5, and suggests the existence of alternative transport systems that compensate for the loss of AMT function in the *qko* mutant at pH 6.5. Influx of ^{15}N-labelled NH_4^+ was measured in roots of 6-week-old plants pre-cultured with either 2.5 mM $(NH_4)_2SO_4$ (Experiment I) or 1.25 mM (Experiment II), and subsequently exposed for 10 minutes to an unbuffered nutrient solution containing 0.1 mM $(NH_4)_2SO_4$. Data represent mean ± SE (n = 5-8). Different letters indicate statistically significant differences among genotypes (one-way ANOVA, Tukey's HSD test, $P < 0.05$). Asterisks (*) denote significant differences relative to the wild-type (Col-0) within the same pH treatment (Student's t-test, $P < 0.05$).
